# A Framework for Managing Device Association and Offloading the Transport Layer’s Security Overhead of WiFi Device to Access Points

**DOI:** 10.3390/s21196433

**Published:** 2021-09-26

**Authors:** Ramzi A. Nofal, Nam Tran, Behnam Dezfouli, Yuhong Liu

**Affiliations:** Department of Computer Science and Engineering, Santa Clara University, Santa Clara, CA 95053, USA; rnofal@scu.edu (R.A.N.); nvtran@scu.edu (N.T.)

**Keywords:** IoT edge computing, TLS offloading, device association, security

## Abstract

Considering the resource constraints of Internet of Things (IoT) stations, establishing secure communication between stations and remote servers imposes a significant overhead on these stations in terms of energy cost and processing load. This overhead, in particular, is considerable in networks providing high communication rates and frequent data exchange, such as those relying on the IEEE 802.11 (WiFi) standard. This paper proposes a framework for offloading the processing overhead of secure communication protocols to WiFi access points (APs) in deployments where multiple APs exist. Within this framework, the main problem is finding the AP with sufficient computation and communication capacities to ensure secure and efficient transmissions for the stations associated with that AP. Based on the data-driven profiles obtained from empirical measurements, the proposed framework offloads most heavy security computations from the stations to the APs. We model the association problem as an optimization process with a multi-objective function. The goal is to achieve maximum network throughput via the minimum number of APs while satisfying the security requirements and the APs’ computation and communication capacities. The optimization problem is solved using genetic algorithms (GAs) with constraints extracted from a physical testbed. Experimental results demonstrate the practicality and feasibility of our comprehensive framework in terms of task and energy efficiency as well as security.

## 1. Introduction

The applications and density of Internet of Things (IoT) stations, also known as IoT devices, are increasing at a very fast pace. It is projected [1] that one trillion new IoT devices (i.e., stations) will be produced by 2035. The number of IoT connections will reach 83 billion by 2024, rising from 35 billion connections in 2020 [2]. In many IoT applications, the stations at the edge are usually constrained in terms of computation and communication resources [3]. There is an unprecedented need for solutions that are more efficient in terms of resource consumption, as these stations become more widely adopted.

Exchanging data with IoT stations requires a secure connection to prevent eavesdropping, tampering, forgery, and other types of attacks. To this end, cryptographic techniques such as public key cryptography (PKC) and symmetric key cryptography (SKC) have been adopted. Secure protocols, such as transport layer security (TLS) and datagram transport layer security (DTLS), are also applied to provide end-to-end communication with authenticity, security, and integrity [4,5]. However, the existing solutions often lead to heavy computation overhead, which IoT stations cannot afford due to their resource-constrained nature. Moreover, a slight increase in a station’s resource consumption causes significantly higher resource consumption when being applied on a large-scale basis across many IoT stations, thereby increasing the energy footprint of IoT technology.

There have been various attempts to reduce the computation overhead of security operations for IoT stations. However, these solutions only partially reduce computation/communication costs, which can be measured in terms of time and energy consumption. In particular, as the amount of data exchanged with stations increases, the overhead of secure communication with stations also increases excessively. Therefore, especially for high-rate standards such as IEEE 802.11 (WiFi) [6], there is a need for a more comprehensive offloading solution to relieve a station’s heavy computation burden by transferring the computation to gateways such as WiFi access points (APs). Given that offloading computation from stations to their associated AP is a classical many-to-one matching problem [7], the question of to which AP a station should be associated becomes significant. An AP has a finite computational capacity, whereas each station requires its associated AP to satisfy its demand. This becomes a device association (DA) problem, a natural extension of station-to-AP computational offloading, and can be defined as an optimization problem with multiple constraints. An optimal solution to the DA problem can ensure a significant reduction in resource consumption for all stations in the entire network. Existing DA studies mainly focus on improving network throughput by considering factors such as signal quality, transmission delay, load balancing, etc. However, these studies seldom consider an AP’s computational capacity a constraint when capacity is actually crucial for identifying the optimal AP to which the extra computation overhead will be offloaded.

This paper treats security offloading and DA as an integrated problem. Specifically, the proposed framework aims to establish lightweight end-to-end secure connections between IoT stations and the cloud by offloading the complex security operations to APs. Furthermore, this study focuses on the scenario where multiple APs are available for a station to be associated with. The goal of DA is to achieve maximum network throughput while utilizing a minimum number of APs. To the best of our knowledge, this is the first study to formulate the DA problem by considering the TLS offloading overhead incurred by security computations. The major contributions of this work are summarized as follows:

- We propose a security offloading framework that allows resource-constrained stations to offload expensive TLS handshake processes to their associated AP securely. This can significantly reduce stations’ resource consumption and improve their lifespan. A testbed is used to implement and evaluate the proposed framework, revealing savings in terms of energy by approximately 15x compared to the conventional approach of establishing TLS handshakes.
- As an integrated component of the offloading framework, we formulate a multi-objective DA optimization problem, aiming to maximize the network throughput via a minimum number of APs while satisfying security requirements and not overloading the APs’ computation and communication capacities. The optimal solution is identified based on genetic algorithms (GAs), which can flexibly support multi-objective functions with constraints. Experimental results demonstrate that the proposed DA scheme can deliver higher throughput compared to other existing DA schemes as the network size grows. Additionally, the proposed DA scheme supports 35% more stations than its closest competitor.

The rest of the paper is organized as follows. Related works are discussed in Section 2. The proposed security offloading framework and DA scheme are discussed in detail in Section 3 and Section 4, respectively. Section 5 presents the experiment setup and analyzes the results. Lastly, Section 6 concludes the study with future research directions.

## 2. Related Work

We categorize the related work into two main groups: (1) offloading schemes and cryptographic optimizations and (2) device association (DA). Table 1 summarizes the characteristics of the most relevant related work.

### 2.1. Offloading Schemes and Cryptographic Optimizations

Offloading the heavy computational security operations is critical to ensure efficient and secure communication and a long lifespan of IoT stations. There has been significant research to provide authentication, privacy, and integrity. For example, in [8], the authors employ GPUs and an optimized implementation of RSA to build a novel IoT architecture, enabling the offloading of only the signature generation component of TLS to a smart gateway. As opposed to this work, our proposed framework offloads the entire TLS handshake, which consists of authentication, confidentiality, and integrity algorithms.

There are a few works on offloading the entire handshaking process of datagram transport layer security (DTLS), which is limited to messages of a 1500-byte size [27]. For instance, the authors in [9] design an architecture that enables resource-constrained devices to establish end-to-end secure communication using DTLS. A dedicated network node is proposed to perform handshake offloading on behalf of an IoT station. The authors in [10] propose to offload DTLS handshake through one trusted gateway. Their work mainly focuses on IEEE 802.15.4, which is not suitable for facilitating communication among large numbers of IoT stations or for large area coverage [28]. In this study, we focus on IEEE 802.11, which has a 300x higher data rate and 10x longer range than the IEEE 802.15.4 standard [9,10,28]. To the best of our knowledge, this proposed framework is the first one to focus on IEEE 802.11 TLS handshake offloading from a resource-constrained IoT station to an AP.

In addition to offloading, another approach is to reduce the computation overhead on stations through algorithmic optimization. Porambage et al. [11] design a lightweight authentication protocol (PAuthKey) to enable mutual authentication and key establishment, providing application level end-to-end security via DTLS with a cipher suite that includes ECDSA and ECDH. In [12], a customized lightweight SSL protocol is proposed; it operates on resource-constrained devices under IEEE 802.15.4 standard and adopts ECDSA and ECDH as its cipher suites. In [13], Zhang et al. were able to improve the efficiency of the RSA algorithm by approximately 50% for a 2048-bit key size to be able to run on a station. Compared to these schemes, the proposed framework reserves only the symmetric algorithm (AES_GCM_256) on the IoT station, resulting in less computational overhead on the stations.

### 2.2. Device Association

In a large network, where multiple APs are available, it is necessary to identify an optimal DA scheme with certain objectives, subject to some constraints. Prior to finding the optimal DA scheme, it is often necessary to consider DA as an optimization problem with various factors. Saad et al. [22] approach the user-association problem in small cell wireless networks by employing analytical techniques based on the college admissions game and coalitional game theory [29,30]. Peng et al. present user to remote radio head association (RRH) strategies for cloud radio access networks (C-RANs) and derive closed-form expressions for the ergodic capacity of the proposed association methods, ultimately providing a theoretical proof of concept [31]. In [15], the authors model DA as a weighted bipartite graph and find the optimal semi-matching using the Kuhn–Munkres (K-M) algorithm. Dandapat et al. frame the DA issue as a max-flow problem and demonstrates that their proposed heuristic is a promising solution [24]. Other works consider the DA problem a mixed/integer linear programming problem [18,19,23]. Existing DA formulations, while novel, mostly incorporate factors such as throughput, signal level, load balancing, channel utilization, link quality, number of transmissions, etc. [15,16,18,19,21,32]. In our study, we treat the AP’s computational capacity as a new major factor, which arises as a natural extension of our offloading framework, along with factors mentioned in the existing studies above. Incorporating the AP’s computational capacity leads us to formulate a multi-objective optimization problem, with the goals of achieving maximum network throughput while requiring a minimum number of APs.

Finally, a DA decision can be made in either a distributed or centralized manner. Distributed mechanisms require stations to collect information on the neighboring APs and to identify an optimal association resulting in extra overhead for stations [23,24,25,26]. This contradicts the purpose of minimizing the computation on IoT stations. On the other hand, our proposed centralized approach takes into account the AP’s computational and communication capacities, as well as security level, as opposed to other studies [15,17,19].

## 3. Secure Offloading Framework

In this section, we introduce TLS basics and the security rationale for the proposed offloading framework. Then, a detailed description of the framework’s functionalities is presented. All handshake functions in this framework refer to the establishment of TLS connections between any two peers.

### 3.1. TLS Preliminaries

In this subsection, we briefly discuss the basics of the TLS protocol to facilitate the understanding of the proposed offloading scheme. TLS consists of two major layers: the handshake protocol and the record layer. The handshake protocol, which adopts PKC, allows the server and the client to authenticate each other and negotiate an agreed-upon cipher suite from a set of related cryptographic algorithms defined for varying needs of security. The record layer, which adopts symmetric cryptography, handles messages between the application layer and the transport layer by performing data fragmentation, encryption, and decryption.

Our previous work [33] has quantitatively measured the significant amount of energy consumption required by these cryptographic algorithms, especially the PKC algorithms and the authentication processes needed by the handshake protocol. Therefore, in this paper, we propose to offload to the AP the asymmetric algorithms as well as signing and verification processes, which usually consume a significant amount of resources [10]. Meanwhile, only the lightweight symmetric encryption will be carried out on the IoT stations based on a pre-shared key, which has been previously distributed by an AP manager to stations and APs, as covered in detail later. We adopt an authenticated encryption with additional data (AEAD) algorithm with the Galois/counter mode (GCM), one of the symmetric ciphers that is recommended in TLS 1.3 [34]. The adopted algorithm can satisfy both security and efficiency requirements at the IoT stations [35,36].

### 3.2. System Overview

This section presents an overview of the proposed framework. In particular, we aim to establish a secure connection between a station and a server on the cloud through the secure TLS handshake protocol. However, rather than performing all the TLS operations at the resource-constrained IoT station end, we propose to offload the TLS handshake component, which is the most computationally heavy process, to the associated AP. This is accomplished by maintaining a secure connection between the station and the associated AP. To achieve this, we mainly consider three types of devices as follows:

1. AP manager (henceforth referred to as mgrGateway): a centralized monitor and controller of the network. It is responsible for real-time station monitoring and station handover as well as dynamic distribution of pre-shared keys.
2. AP: a set of *m* access points marked as $\left\{ap_{1},ap_{2},\cdots \;ap_{m}\right\}$, all working on the same channel to facilitate connections between stations and the cloud. Every station associated with an AP is allocated airtime in a manner that ensures each station’s demand is met. We also assume that only one station can transmit at a given time for every AP. The order of airtime allocation follows the heuristic approach in which the station with maximum demand transmits first.
3. STA: a set of *n* IoT stations marked as $\left\{sta_{1},sta_{2},\cdots \;sta_{n}\right\}$.

**Figure 1:**
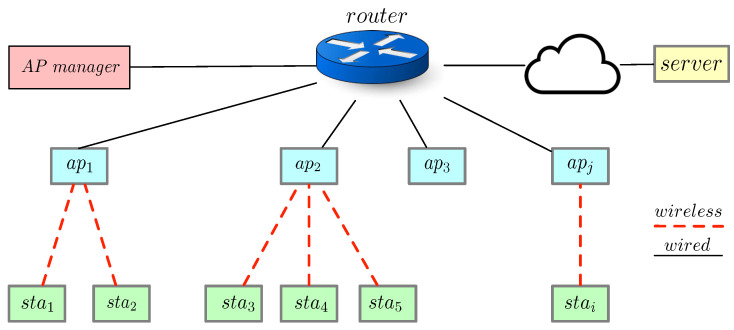
The overall system architecture. Stations are connected with their associated APs. APs are connected to an *AP manager* through one or multiple routers.

Figure 1 depicts the architecture of the framework, showing interactions among different devices in the network. All APs share the same SSID and passphrase. When a station enters the network, it needs to first connect to the mgrGateway, through which it will be associated with an appropriate AP. Afterwards, when the station needs to establish an end-to-end secure connection with a server on the Internet through TLS, it will offload the TLS computation to its associated AP, which forms a link between the AP and the mentioned server. The other part of the connection is between the station and the AP. Such a connection uses the symmetric key that is dynamically distributed when that station joins the network and becomes associated with that AP, as shown on the left side of Figure 1. The key idea is that the station only needs to undergo the TLS process once with mgrGateway. From that point onward, the station can access any desired server on the Internet through the associated AP without needing to perform the TLS handshake again. The core advantage of the proposed architecture is that stations can offload computation to the APs, freeing precious resources for other computational tasks. Note that the communication between each station and its associated AP are also secured by the layer-2 WPA2 or WPA3 method of WiFi [6].

### 3.3. Security Analysis

Whenever an AP or a station joins the network it is required to establish a TLS handshake with the mgrGateway. A certificate is required for each node during the TLS handshake for mutual authentication. Furthermore, through the TLS session with mgrGateway, a symmetric 256-bit key is generated and subsequently used to secure later message exchanges between mgrGateway and the other node. In this case, the other node is the AP or station that has recently joined the network. The usage of symmetric keys between the node pairs is described in detail in the sequence diagrams later in the paper.

To facilitate these connections, the key security parameters are stored in different tables managed by mgrGateway or APs. In particular, mgrGateway manages the following tables: apTable, staTable, and mgrKeyTable. These tables store and maintain real-time information about all network nodes. In particular, apTable shows information about individual APs that are available at any given time in the network, including $SK_{gj}$, which is the shared key between mgrGateway and any AP $ap_{j}$. Furthermore, staTable has information on individual stations present at any given time in the network, including the IP and MAC address of the station. In mgrKeyTable, the key $SK_{ji}$ (shared key between station $sta_{i}$ and AP $ap_{j}$) is also saved, in case mgrGateway moves $sta_{i}$ to a different AP. Finally, each AP (i.e., $ap_{j}$) manages its own apKeyTable, which shows all the stations connected to it and their associated shared keys.

The proposed framework can effectively defend against different types of prevalent exploits in IoT settings, including man-in-the-middle (MITM), eavesdropping, packet manipulation, replay, and known-key [37]. The proposed framework is secured against the MITM exploit by the mutual authentication provided by TLS for any communications between a station/AP and the mgrGateway [38]. For eavesdropping attacks, even if an eavesdropper manages to capture any packets, the encryption mechanism ensures that the eavesdropper cannot extract any meaningful data from the sniffed packet [37]. This is because each packet is either part of the end-to-end TLS session or protected by a previously distributed pre-shared key between a station and its associated AP.

Packet manipulation is also rendered challenging by the framework, as every packet is secured by an AEAD encryption mechanism, such that any tampering can be detected by the receiver. On the other hand, if an attacker tries to replay a packet, the replayed packet will be rejected by the receiving party, because every message sent using the GCM algorithm includes both an encoded nonce and a counter in the AEAD. A replayed packet will cause different counters at the sender and the receiver ends, resulting in a failed integrity check [37]. Last, but not least, the proposed framework is robust against known-key attacks, as the framework enforces the use of ECDHE for key exchange, featuring forward secrecy. Even if a malicious entity compromises a session key, it is not able to decrypt the previous sessions.

### 3.4. Adding an AP

**Figure 2:**
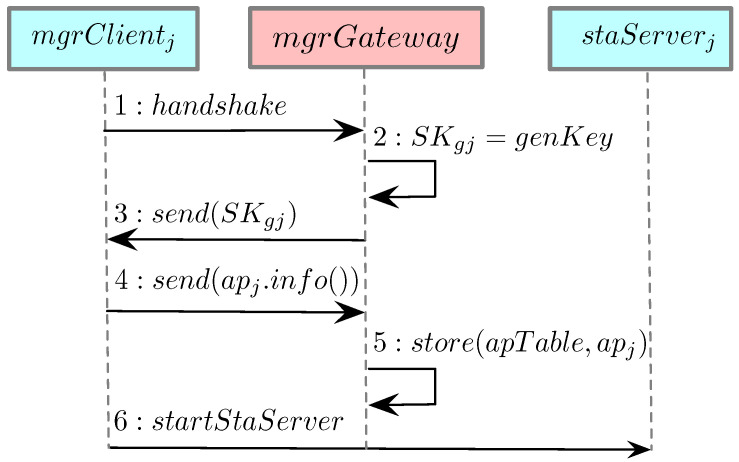
Sequence diagram describing the process of adding a new AP to the network. Once added, the new AP can be used for computational offloading.

This subsection covers the process of adding a new AP to the network, as shown in Figure 2. In this particular scenario, two nodes are involved: the mgrGateway and the new AP joining the network. The new AP needs to play two types of roles. The first role is as a client to connect with mgrGateway, which is marked as $mgrClient_{j}$. The second role is as a server (i.e., marked as $staServer_{j}$) to provide offloading services to potential stations connecting with it in the future. Therefore, as shown in Figure 2, both $mgrClient_{j}$ and $staServer_{j}$ are marked in the same color to indicate two different roles played by the new AP.

Upon booting up, the AP typically requests to join the network and executes the client code $mgrClient_{j}$, where *j* represents $ap_{j}$. Subsequently, the process of adding an AP occurs as follows: **Step 1**: A TLS handshake is performed between $mgrClient_{j}$ and mgrGateway to establish a secure channel for the remaining steps. The handshake involves mutual certificate authentication. **Step 2**: The mgrGateway generates a shared key $SK_{gj}$ (where *g* represents the mgrGateway). **Step 3**: The mgrGateway sends the shared key $SK_{gj}$ to the $mgrClient_{j}$. This shared key will be used between mgrGateway and the new AP $ap_{j}$ for all the messages exchanged between them henceforth. **Step 4**: $mgrClient_{j}$ sends its information to the mgrGateway: MAC address $ap_{j}^{mac}$ and an available port. The port will be used later to create socket connections with IoT stations. **Step 5**: mgrGateway stores the values of $ap_{j}^{ip}$ ($ap_{j}$’s IP address previously extracted from the TLS handshake), $ap_{j}^{mac}$, $SK_{gj}$, and the available port in apTable. **Step 6**: At last, the $mgrClient_{j}$ forks a new program called $staServer_{j}$, that will serve IoT stations associated with this AP. $ap_{j}^{mac}$ is used to establish a wireless connection between stations and $ap_{j}$, while $ap_{j}^{ip}$ and the available port are used to establish a socket connection. In addition, it is $staServer_{j}$ that will later carry out the offloaded functions for all associated stations.

### 3.5. Adding a Station

**Figure 3:**
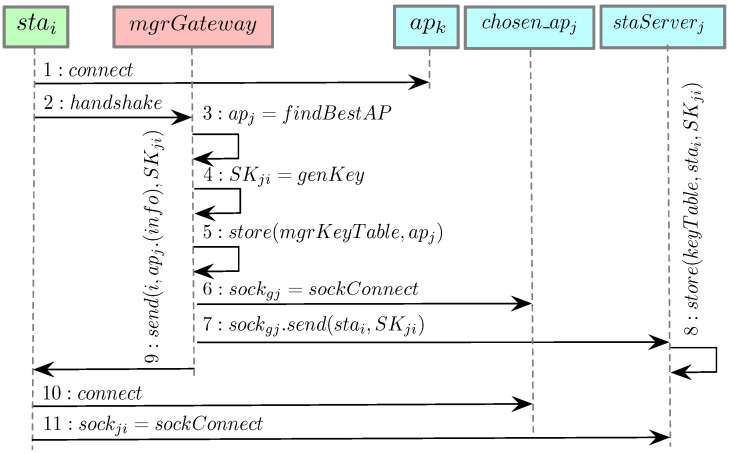
Sequence diagram describing the process of adding a new station to the network. During this process, a newly joined station is associated with an optimal AP. Once this process is finished, a station can offload its TLS computation to the associated AP.

This subsection explores the procedure of adding a new station to the network, as shown in Figure 3. A station, shown as $sta_{i}$, obtains credentials and interacts with the AP and mgrGateway. The AP encompasses the following entities in the sequence diagram: $ap_{k}$, the chosen $ap_{j}$, and $staServer_{j}$. Adding a new station to the network happens as follows: **Step 1**: When a new station first seeks to join the network, it arbitrarily connects to any AP available (e.g., $ap_{k}$). **Step 2**: Based on step 1, a TLS handshake can then be established between the station $sta_{i}$ and the AP manager mgrGateway. Information related to this station (e.g., IP) is stored and/or updated in staTable. The handshake involves mutual certificate authentication, preventing malicious stations from joining the network. **Step 3**: Based on the proposed DA scheme, mgrGateway identifies the optimal AP $ap_{j}$. The details of the proposed DA scheme will be discussed in Section 4. At this point, mgrGateway retrieves the information about the selected $ap_{j}$ from apTable and returns information about $ap_{j}$ to the station $sta_{i}$. This information is collected whenever a new AP initially joins the network, as demonstrated by Figure 2. **Step 4**: mgrGateway creates a symmetric shared key $SK_{ji}$, which will be used between $sta_{i}$ and $ap_{j}$. **Step 5**: mgrGateway generates and stores information about $sta_{i}$, $ap_{j}$ as well as their shared key $SK_{ji}$. These values can be used later when mgrGateway hands stations over to different APs to balance the network. **Step 6**: mgrGateway establishes a socket connection $sock_{gj}$ (where *g* represents the mgrGateway) with $ap_{j}$ using $ap_{j}^{ip}$ and the corresponding port, retrieved from apTable. **Step 7**: mgrGateway also locates the shared key $SK_{gj}$ from apTable, which will be used to send and receive encrypted messages between mgrGateway and $ap_{j}$. Then, mgrGateway sends a message containing $sta_{i}$ and $SK_{ji}$ to $ap_{j}$’s server module $staServer_{j}$. **Step 8**: Upon receipt, $staServer_{j}$ stores $sta_{i}$ and $SK_{ji}$ in apKeyTable. **Step 9**: Through the secure channel established between the AP manager and the station $sta_{i}$, mgrGateway sends the following values to $sta_{i}$: $SK_{ji}$, $ap_{j}^{ip}$, $ap_{j}^{mac}$, and the port. **Step 10**: $sta_{i}$ connects to $ap_{j}$ using $ap_{j}^{mac}$. **Step 11**: $sta_{i}$ establishes a socket connection to $staServer_{j}$ using $ap_{j}^{ip}$ and the previously retrieved port. This creates an association between $sta_{i}$ and $ap_{j}$. Later, when $sta_{i}$ connects to any server on the Internet, $ap_{j}$ will facilitate the connections by carrying out the TLS handshake with the remote cloud servers. The station only needs to connect to $ap_{j}$ through a secure channel using $SK_{ji}$.

### 3.6. Station Handover

**Figure 4:**
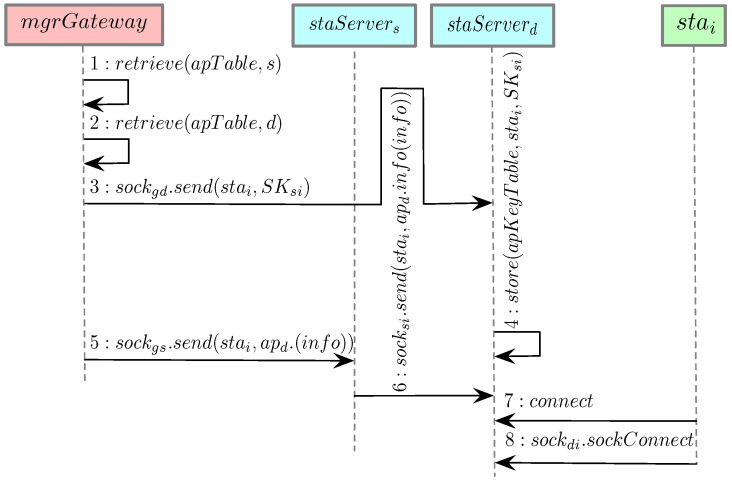
Sequence diagram of station handover functionality. This process is used to hand a station over to another AP.

When a station moves or a particular AP reaches its capacity, it is possible for one or multiple stations to be handover to a different AP. This subsection describes the steps needed to move station $sta_{i}$ from a source AP $ap_{s}$ to a destination AP $ap_{d}$, as shown in Figure 4. This process involves the AP manager mgrGateway, the station $sta_{i}$, and the server module of the source and destination APs $staServer_{s}$ and $staServer_{d}$.

**Step 1 and 2**: mgrGateway first retrieves information from apTable about two APs: $ap_{s}$ and $ap_{d}$. This information includes: two previously stored sockets ($sock_{gs}$ and $sock_{gd}$), three shared keys ($SK_{gs}$, $SK_{gd}$, and $SK_{si}$), and other information (IP, and MAC address and port for both APs). It should be noted that *s*, *d*, *g*, *i* represent $ap_{s}$, $ap_{d}$, mgrGateway, and $sta_{i}$, respectively. **Step 3**: mgrGateway sends a message encrypted by the shared key $SK_{gd}$ through the socket $sock_{gd}$ to $staServer_{d}$. This message contains information about $sta_{i}$ and the shared key $SK_{si}$ which has been previously used between $sta_{i}$ and $ap_{s}$. $SK_{si}$ is now used to encrypt subsequent messages between $sta_{i}$ and $staServer_{d}$. **Step 4**: The $staServer_{d}$ stores the recently received information about $sta_{i}$ as well as $SK_{si}$ in apKeyTable. **Step 5**: mgrGateway uses socket $sock_{gs}$ and shared key $SK_{gs}$ to send a message to $staServer_{s}$ with the following content: $sta_{i}$, IP address, MAC address, and port of $ap_{d}$ (all previously obtained in steps 1 and 2). **Step 6**: The $staServer_{s}$ uses $sock_{si}$ and $SK_{si}$ to propagate the message from the previous step to $sta_{i}$, asking $sta_{i}$ to connect to $staServer_{d}$. **Step 7**: Upon receiving the message from the previous step, $sta_{i}$ connects through WiFi to the AP $staServer_{d}$. **Step 8**: $sta_{i}$ establishes a socket connection to $staServer_{d}$. Any subsequent messages between $sta_{i}$ and $staServer_{d}$ will be encrypted by $SK_{di}$ (previously known as $SK_{si}$). It should be noted that $sta_{i}$ has not changed the shared key between itself and $staServer_{d}$. At this point, $sta_{i}$ is handed over from source AP $ap_{s}$ to destination AP $ap_{d}$.

### 3.7. TLS Offloading

With the previously discussed modules, the proposed framework is able to offload the TLS handshake protocol, which is the most computationally expensive component of TLS, from IoT stations to their associated APs.

To begin the offloading procedure, the station $sta_{i}$ associated with the AP $ap_{j}$ performs a series of tasks. First, $sta_{i}$ sends a request $req_{ic}$ to $ap_{j}$. This request is encrypted using $SK_{ji}$ between $sta_{i}$ and $ap_{j}$; the request contains information about the cloud server *c* to which the station seeks to send and receive messages. Second, after receiving this encrypted request, $ap_{j}$ decrypts it using the same shared key $SK_{ji}$ and performs a handshake with the server *c*. Third, $ap_{j}$ sends the extracted content from the request $req_{ic}$ to the server *c* and obtains a response, $res_{ic}$. Finally, $ap_{j}$ encrypts the response $res_{ic}$ with $SK_{ji}$ and sends the encrypted response back to $sta_{i}$. Such offloading can significantly reduce the resource consumption on the IoT stations while satisfying the security requirements.

## 4. Device Association

In an extensive network where multiple APs are available, an IoT station can be associated with different APs, requiring a process to identify the most appropriate AP. We define such a device association (DA) process as an optimization problem of mapping *n* stations to no more than *m* given APs. The maximum network throughput can be achieved with the minimum number of APs involved. More importantly, we assume that each AP has limited capacity to facilitate offloading and set such limitations as constraints in our problem formulation. Such an assumption differentiates this work from most existing ones. Furthermore, finding the best association is an NP-hard problem. To address this problem, we propose an efficient DA scheme that can adapt to the dynamics of large-scale networks.

### 4.1. Association Problem Formulation

We formulate the constraints for optimized association using several backbone formulas and quantities. In this study, the stations usually operate in the context of IoT and thus transmit messages reporting different status types to the AP. Each station provides information about its demand when it first joins the network. Such demand remains fixed throughout a station’s lifespan.

In particular, we mathematically formulate the optimization model as a multi-objective problem involving two components: (1a) maximizing the total network throughput, and (1b) minimizing the number of active (required) APs. The key symbols are summarized in Table 2.

(1a)maximize ∑i=1n∑j=1mlog(1+rji∗cji)(1b)minimize ∑j=1maj(1c)subject to:∑j=1mcji=1     ∀i∈STA(1d)    ∑j=1m(ψji∗cji)≥P0  ∀i∈STA(1e)    ∑j=1m(Rji∗cji)≥R0 ∀i∈STA(1f)    ∑i=1nsli∗cji≤S0   ∀j∈AP(1g)    ∑k∈K∑i=1nmik∗cjif(mk)≤1  ∀j∈AP

There are multiple constraints considered in the model. The constraint (1c) ensures that every station is associated with exactly one AP. The constraint (1d) guarantees that the signal received from any station has to be above a certain threshold $P_{0}$ in order for the AP to sense and process that signal, as dictated by IEEE 802.11 standards. The constraint (1e) makes sure that the received signal by the station from the AP has to be above a certain threshold $R_{0}$ (minimum RSSI threshold) in order to ensure proper transmitting and successful association. These are typical constraints considered by existing studies for device association.

Beyond the above constraints, we propose a security constraint (1f) to control the maximum number of stations transmitting sensitive data that an AP can serve. We assume that a station $sta_{i}$ may have its own security level requirement (i.e., marked as $sl_{i}$). A higher value of $sl_{i}$ indicates a higher security requirement and thus more computation complexity. The sum of these quantities across all stations served by an individual AP cannot exceed a certain threshold, $S_{0}$, which can be customized based on the network’s security conditions. A smaller $S_{0}$ value can help limit not only the security overhead on each AP, but also the number of stations associated with a single AP. Therefore, in case the AP is compromised, fewer stations are impacted.

We propose the constraint (1g) to consider the APs’ computation and communication capacities. In particular, we consider that different types of messages (i.e., represented by *k* with size $m^{k}$) may require different computation/communication resources. The function *f*, which returns the maximum number of messages that can be processed by an AP per second, can be implemented as either $f_{sc}$ or $f_{c}$ to represent different scenarios. Specifically, the function $f_{c}$ mainly focuses on plaintext messages that are not encrypted. On the other hand, the function $f_{sc}$ mainly focuses on encrypted messages for secure communications. Since additional computation is required for continuous encryption and decryption, the AP can only process a smaller number of messages, leading to smaller return values for $f_{sc}$ when the inputs are the same. Both of these functions are customized functions. Therefore, in this study, we quantitatively evaluate their function based on a real testbed, which is discussed more in Section 5.

Furthermore, some key parameters involved in these constraints are calculated as follow: first, the received signal strength indicator (RSSI) at $sta_{i}$, represented by $R_{ji}$, is calculated by subtracting the total path loss, measured in dB, from $ap_{j}$’s transmission power $P_{T_{x}}^{j}$ [39]. On the other hand, $\psi_{ji}$ refers to the received signal strength at $ap_{j}$ from station $sta_{i}$, which is calculated by subtracting the total path loss from $sta_{i}$’s transmission power $P_{T_{x}}^{i}$. Without losing generality, we simply assume that all nodes in the network have the same transmission power and use omnidirectional antennas. The total path loss is calculated as $L_{D_{0}}+10\gamma \log_{10}\left(\frac{d}{D_{0}}\right)+X_{g}$, where $L_{D_{0}}$ represents the path loss at a reference point $D_{0}$; γ is the path loss exponent; and $X_{g}$ is a zero-mean Gaussian distributed random variable (in dB) [40]. It should be noted that $X_{g}$ is used only when there is a shadowing effect, and will be set as zero if the shadowing effect is not considered.

Second, we calculate the transmission rate between the station $sta_{i}$ and the AP $ap_{j}$ (marked as $r_{ji}$). Based on the Shannon–Hartley theorem [40], the transmission rate $r_{ji}$ can be calculated by multiplying the binary logarithm of ${\mathrm{SNR}}_{ji}$ with B, the channel bandwidth. In reality, the actual rate may also be affected by the chosen modulation and coding scheme (MCS).

(2) $$r_{ji}=B\log_{2}\left(1+{\mathrm{SNR}}_{ji}\right)$$

Specifically, for a given uplink connection from $sta_{i}$ to $ap_{j}$, ${\mathrm{SNR}}_{ji}$ of the $ap_{j}$ can be calculated as

(3) $${\mathrm{SNR}}_{ji}=\frac{\psi_{ji}}{I\left(ap_{j},sta_{i}\right)+\sigma^{2}}$$

In Equation (3), $\sigma^{2}$ refers to the value of additive white Gaussian noise (AWGN). The function $I(ap_{j},sta_{i})$ returns the cumulative interference produced by all other transmitting stations that interfere with station $sta_{i}$ for a given $ap_{j}$. ‘Transmitting stations’ refers to all stations in the entire network, including those associated with other APs.

The function $I(ap_{j},sta_{i})$ is described in detail by Algorithm 1 where $ap_{j}$ and $sta_{i}$ are the input ($sta_{i}$ is associated with $ap_{j}$) and the interference value is the output. Given an arbitrary station $sta_{u}$, associated with any AP $ap_{z}$ such that j≠z, if $sta_{u}$ interferes and transmits concurrently with $sta_{i}$, then $g_{ui}$ is 1, else $g_{ui}$ is 0. When $g_{ui}$ is 1, the interference value is incremented by $\psi_{zu}$.
**Algorithm 1:** Procedure describing function $I(ap_{j},sta_{i})$ in Equation (3)
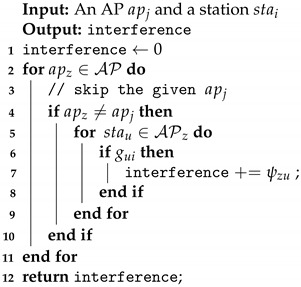


**Figure 5:**
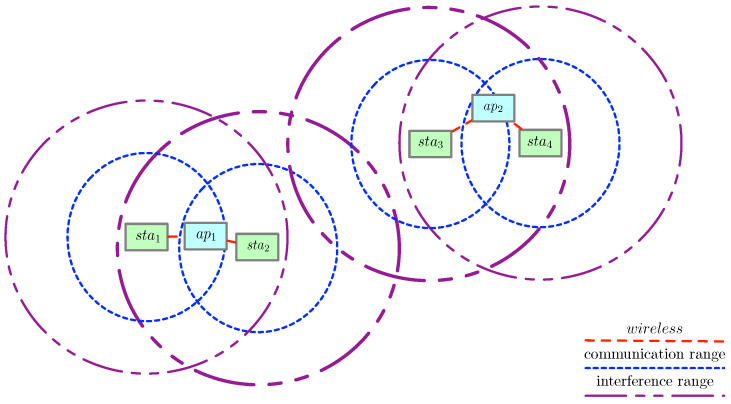
Communication and interference ranges for stations in a simple topology.

An illustration of our assumed communication and interference model is shown in Figure 5. As an example, Algorithm 1 can be used to compute the interference of ${\mathrm{SNR}}_{12}$ between $sta_{2}$ and $ap_{1}$. Assuming that $g_{23}$ equals 1, meaning $sta_{2}$ and $sta_{3}$ are interfering with each other and transmitting at the same time, the function I(1,2) returns $\psi_{23}$. Therefore, the denominator of ${\mathrm{SNR}}_{12}$ is $\psi_{23}+\sigma^{2}$. As another example, we compute the interference of ${\mathrm{SNR}}_{24}$ between $sta_{4}$ and $ap_{2}$. In this case, the function I(2,4) returns 0 as none of the stations from $ap_{1}$ interferes with $sta_{4}$, therefore the denominator of ${\mathrm{SNR}}_{24}$ is only $\sigma^{2}$.

The interference calculated by the function $I(ap_{j},sta_{i})$ can be treated as AWGN. The reason is that the interference is not dominated by only a few interferers. For example, if every AP has one station associated, the interference per station will be impacted by all the stations in the network, as all stations will be transmitting concurrently. On the other hand, in a network with only one AP and multiple associated stations, there is zero interference from other stations when a station transmits.

Once ${\mathrm{SNR}}_{ji}$ is determined, we can use the Shannon–Hartley theorem to calculate $r_{ji}$. To formulate our first objective (1a), used as a common quantitative measure for DA scheme comparison, we employ $r_{ji}$. The second objective (1b) of our DA optimization problem is to minimize the number of APs needed. The resulting resource consumption reduction on both stations and APs is significant, as measured in the form of energy and time later in Section 5.

### 4.2. Proposed DA Scheme

In this study, we adopt genetic algorithms (GAs), an effective approach to resolve optimization problems, to associate stations with APs. This method allows us to add a wide range of dynamic network constraints flexibly to yield a well-optimized association solution. Some of the classic problems that GAs can solve effectively include bin allocation, knapsack, and traveling salesman [41,42,43]. To the best of our knowledge, this is the first study to adopt GAs to address the WiFi station association problem. In particular, we revise the default GA by setting the optimization goal as maximizing network throughput and minimizing the number of APs. Furthermore, several core functions of the GA, including *fitness, crossover*, and *mutation*, have been revised to fit our specific device association needs.

The basic working mechanism of the GA is as follows. Inspired by how genetics work, a GA program begins with a set of variables that internally resemble the chromosomes storing human genetic information. This involves an initial set of individuals representing candidate solutions. A feasibility function checks if an individual satisfies a list of constraints before declaring it valid. Invalid individuals are disposed of, while valid individuals’ **fitness values** are evaluated. For every generation, either crossover or mutation occurs at a configurable probability. **Crossover** occurs on valid individuals by the mate function to create child individuals for the next generation. Additionally, a certain number of individuals are subject to **mutation**, a process that helps add more diversity into the system by producing more interesting individuals. Finally, only a small percentage of the entire valid population is selected for the next generation using the select function, which chooses the individuals that best satisfy an objective function out of the subset mentioned above. The entire process is repeated for a given number of generations. Ultimately, the entire population evolves, but only the best individuals are selected as the final solution in the hall of fame, which keeps track of the individuals with the greatest fitness at any given time. Pareto efficiency is used as the criterion to select the best individuals for the hall of fame. One crucial characteristic, and perhaps weakness, of GAs is that there has to be a clear way to evaluate the fitness of a potential solution.

To adopt the GA, we format the association problem modeled in Section 4.1 by representing each candidate association solution (i.e., individual in the GA) as a binary matrix. In this matrix, each row represents a specific AP, and each column represents a specific station. If an entry at (j,i) is 1, it means $sta_{i}$ is connected to $ap_{j}$. In this matrix format, a specific candidate association solution can be easily compared and evaluated for fitness.

**Figure 6:**
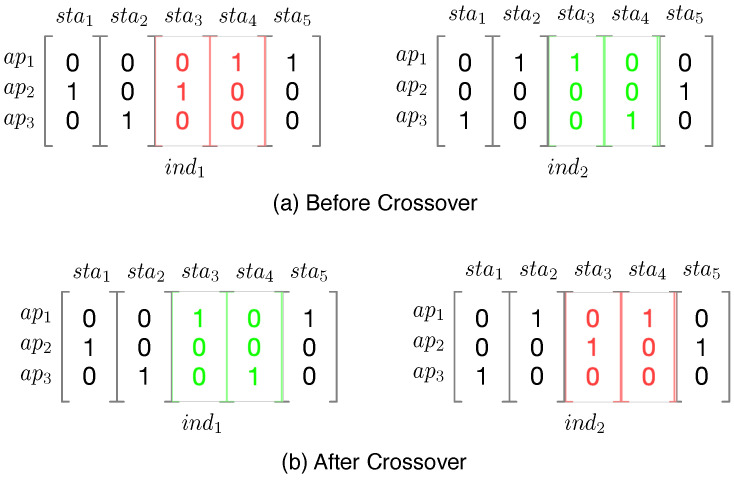
Crossover process. $ind_{1}$ and $ind_{2}$ are two input individuals, which are transformed by the crossover function. Here, $sta_{3}$ and $sta_{4}$ are chosen at random to be swapped during crossover to create new individuals.

We illustrate the crossover process implemented in this study in Figure 6. Specifically, during the crossover, two individuals, which are binary matrices, are considered as inputs. Let us consider the crossover operation on two individuals, called $ind_{1}$ and $ind_{2}$, respectively. During this process, a particular range of consecutively numbered stations is chosen at random, which are then swapped between both individuals. For example, if $sta_{3}$ and $sta_{4}$ are chosen to be swapped, then at the end of the crossover process, the two individuals will be transformed through the swapping of columns ($sta_{3}$ and $sta_{4}$) and become two new individuals.

**Figure 7:**
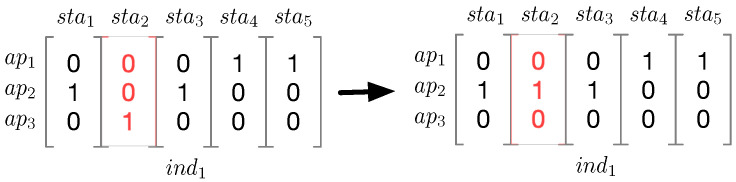
Mutation process. This shows the mutation of an individual, namely $ind_{1}$. $sta_{3}$ is chosen at random to change its associated AP during mutation.

In addition, we also tailor the mutation function for our association problem, as depicted in Figure 7. During mutation, which takes an individual as an input, a station is chosen at random to be associated with another AP, which is also randomly selected. For example, an $ind_{1}$ can experience a mutation, in which $sta_{2}$ is selected through randomness to change its associated AP.

The purpose of crossover and mutation is to introduce more randomness into the population to ensure that the evolutionary process is not trapped in a suboptimal solution.

## 5. Experiments and Results

This section discusses the testbed setup and the experiments for performance evaluation of the proposed framework, along with results and analysis.

### 5.1. Specifications of Network’s Nodes

Table 3 summarizes the specifications of the nodes present in the network. The three platforms used in the network are CYW43907, Raspberry Pi 4, and an Intel Core i5 machine. First, this study uses multiple CYW43907 (CYW) [44] boards as stations. CYW is an embedded wireless system-on-a-chip (SoC). Boasting the powerful ARM Cortex-R4 processor and an on-chip cryptography core, the CYW board is optimized for IoT computation-heavy applications and supports hardware-accelerated AES. The development on the CYW platform is carried out in C code using WICED Studio version 6.1.0 [45], the standard SDK for the CYW platform. This SDK includes a free, open-source library of cryptographic algorithms for embedded systems called mbed TLS [46].

Second, multiple RPi4s and Intel_i5 machines are adopted as APs. Raspberry Pi 4 (RPi4) is a single-board computer and features strong computing power with support for a variety of communication standards [47]. At the time of writing, RPi4 is not known to support hardware-accelerated cryptography natively. To diversify the testing platforms, a machine using Intel Core i5 (Intel_i5), which has more computational resources than RPi4, is also included. This ensures a variety of hardware during our study, as these high-performance platforms are convertible to an AP (this can be carried out by using hostapd, a user-space daemon enabling a host to act as an AP) and powerful enough to handle computation offloaded by the stations. For development on the APs, Python is chosen as it allows for quick prototyping.

Third, the AP manager mgrGateway is hosted on either an RPi4 or an Intel_i5 to enable simulations with larger networks and higher performance requirements.

Last, but not least, to achieve high speed transmission, we use a high-performance WiFi dongle BrosTrend AC1200 USB WiFi Network Adapter (5GHz). The guaranteed speed between the AP and the station is measured by the iPerf utility to be between 240 to 300 Mbps.

### 5.2. Energy and Time Measurement Tool

This work uses a powerful evaluation tool, EMPIOT, developed by our previous work [48] for energy and time measurement of IoT stations. EMPIOT is a shield board installed on top of a Raspberry Pi. The start–stop mechanism of EMPIOT energy measurements can be carefully controlled by utilizing the GPIO pins of the Raspberry Pi. EMPIOT is accurate to 0.4 μ W when measuring energy. When taking measurements on IoT devices using 802.15.4 and 802.11 wireless standards, the EMPIOT’s energy measurement errors are less than 3%. When using 12-bit sampling resolution, this tool can stream 1000 samples per second. All energy and time measurements in this study have been carried out using this platform.

### 5.3. Reduction of Resource Consumption via Offloading

The proposed offloading framework is able to deliver substantial reductions in resource consumption on an IoT station. In order to evaluate the duration and energy consumption on the CYW board, we conducted a series of experiments in which a CYW board and an RPi4 serve as a station and a server, respectively. The tasks executed on the station correspond to the columns in Table 4 as follows: (1) **HS**: establishing a TLS handshake, (2) **HS and records**: establishing a TLS handshake and sending 512 messages (each of 16 bytes) to the server, and (3) **encrypted messages**: establishing a TCP socket, sending two 4096-byte messages encrypted using symmetric keys to the server and closing the socket.

As shown in Table 4, **HS** demands a significant amount of time and energy. Therefore, sending messages on top of establishing a TLS connection requires even more resources, as depicted in column **HS and records**. However, the resource consumption is significantly reduced by adopting our proposed offloading framework, which uses TCP to create a socket for secured communication with symmetric cryptography. Column **Encrypted messages** reveals that our offloading approach uses 15 times less energy than the conventional approach shown in column **HS and records**.

In our proposed offloading framework, a station needs to make at most one TLS handshake, **HS**, with mgrGateway. Next, the station is assigned to an AP using our proposed DA scheme. From that point onward, all communication between the station and the cloud occurs through the AP with a TCP socket, which is very economical as seen in column **Encrypted messages**. In contrast, for a conventional network, a station would have to establish a TLS connection directly with a server on the cloud every time there is a need for communication. Column **HS and records** reveals that this conventional approach is, computationally, highly expensive.

Earlier works, such as [8], propose offloading the signature calculation of the TLS handshake. Based on our previous empirical analysis of TLS resource consumption [33], which uses a similar physical testbed to this study, a single 2048-bit RSA signature calculation performed on a CYW board requires 0.169J of energy and 0.201 s. If this signature calculation were to be offloaded to another node in the network, based on subtracting the mentioned signature generation values from those of column **HS**, the resource consumption on the station would be 2.369J and 20.207s. Even with such savings, the resource consumption remains high as compared to our proposed framework consumption (column **Encrypted messages**).

### 5.4. Estimating AP Capacity via a Real Testbed

**Figure 8:**
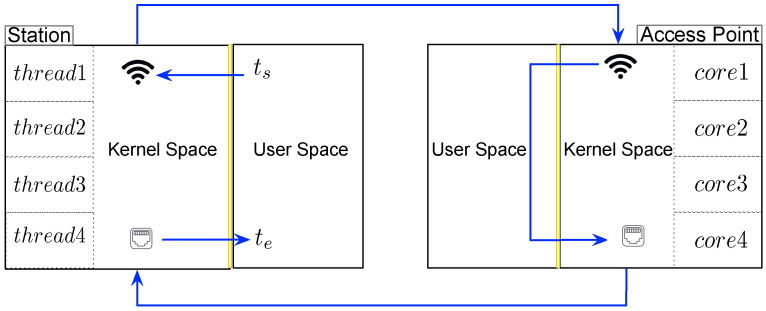
The closed-loop testbed used to determine $f_{c}$ and $f_{sc}$. At the time $t_{s}$, a packet is originated from the station and is sent to the AP. Once this packet is received, a new packet is created on the AP and sent to the station, completing a closed loop at time $t_{e}$.

This subsection presents the proposed closed-loop testbed, which quantitatively measures the AP’s message processing capacity. As shown in Figure 8, the proposed testbed mainly includes two devices: an AP and a station. In particular, we employ both an ARM-based platform (RPi4) and an Intel-based machine (Intel_i5) to diversify the experiment settings. Furthermore, since the transmission delay between the AP and the cloud server is not the focus of this study, we close the loop in our testbed by directly connecting the AP back to the station through an Ethernet connection, for which the transmission delay can be ignored.

The software components of the testbed include several applications running on the station and the AP to transmit and receive the messages. Since the AP has four cores, four threads are created at the station to send packets through the wireless channel to ensure that the AP utilizes its maximum processing capacity. Another four threads are created at the station to receive responses through the wire.

Given a specific packet size, an AP’s processing capacity is measured as the number of packets it processes per second per core, represented by the outputs of $f_{c}$ for plaintext packets and $f_{sc}$ for encrypted packets. To this end, it is necessary to measure the time spent by the AP’s CPU to process a message for a duration marked by $t_{s}$ and $t_{e}$. Tracing the arrows in Figure 8 allows us to understand the path of the message. At the time $t_{s}$, a message starts its path at the user-space of the station. It then proceeds to the kernel space of the station and is transmitted to the kernel space of the AP using WiFi. After traversing the AP’s user space and kernel space, the message is sent through an Ethernet wire back to the station. This completes the closed loop and is marked by the timestamp $t_{e}$.

Since the AP’s CPU is working close to 100% capacity, the duration marked by $t_{s}$ and $t_{e}$ includes context switches between kernel space and user-space as well as encryption and decryption (if the ciphertext is used) along the way. This duration can be used to find how many messages can be processed per second. By repeatedly determining such duration for different sizes/types of messages across different platforms, we are able to estimate $f_{c}$ and $f_{sc}$ for different inputs.

**Figure 9:**
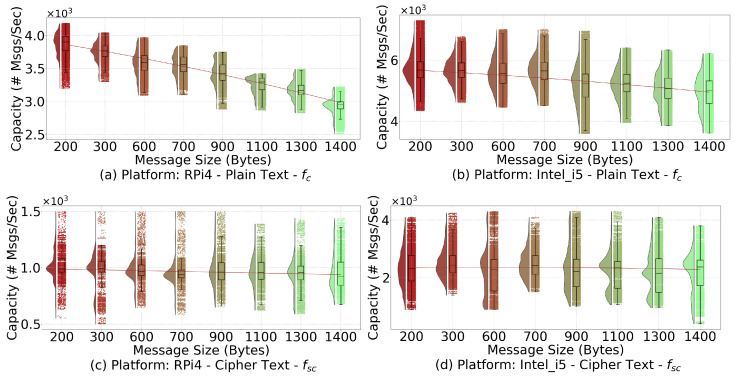
Raincloud plots for $f_{c}$ and $f_{sc}$ on two platforms: RPi4 and Intel_i5. The regression lines from these plots enable us to determine $f_{c}$ and $f_{sc}$ using the message size as input.

Figure 9 shows the plots for $f_{c}$ and $f_{sc}$ when running the testbed with RPi4 and Intel_i5 platforms serving as APs. The data points are shown along with the corresponding boxplots and distributions in the form of half-violins to the left of each grouping. The plotting follows an approximate Gaussian distribution for all the groupings. In this figure, there is a downward trend for both subplots showing $f_{c}$. The number of messages processed per second goes down in a pure communication setting with no encryption/decryption as the message size increases. However, for subplots depicting $f_{sc}$, the trend is more or less uniform. Different patterns indicate that the packet transfer component cannot efficiently handle all the messages when their sizes increase significantly. In other words, the bottleneck to process messages at the AP is not the encryption/decryption operation but rather the packet transfer component.

The actual coefficients of the regression functions for $f_{c}$ and $f_{sc}$ are summarized in Table 5. In the later experiments, we select the regression lines from both the Intel_i5 and RPi4 plots in Figure 9 to estimate the $f_{c}$ and $f_{sc}$ functions.

### 5.5. Comparison Schemes

In this study, we use the Distributed Evolutionary Algorithms in Python library (DEAP) [49] to implement the proposed GA scheme. A complete summary of the key parameters used by the GA is provided in Table 6. These parameters are fine-tuned to achieve the best performance by following the recommendations from DEAP’s documentation.

Furthermore, for performance validation, we compare the GA with the following four algorithms:

- Round robin (RR): A station selects the next AP for association in a round robin fashion. If the station is not able to associate with the selected AP, the algorithm returns.
- Received signal strength indicator (RSSI): A station selects an AP with the strongest signal indicator for association. A heuristic for signal strength is the distance between the station and the AP. If the station is not able to associate with the selected AP, the station selects the AP with the next strongest signal indicator. This process continues until either association occurs or no AP can satisfy the station’s demand, the latter causing the algorithm to return.
- User decision (UD): A station is associated with a user-chosen AP. This approach is commonly used in practice. For this study, UD is implemented such that every station selects an AP at random from a set of APs that are able to satisfy the station’s demands. If the set is empty, the algorithm returns.
- Mixed integer linear programming (MILP): Stations are associated with APs based on the solution to a mixed integer linear programming problem.

Except for MILP and the GA, the remaining algorithms are iterative approaches for which the order of association is crucial. We use both iterative and non-iterative methods as benchmarks to validate the performance of our proposed DA scheme. MILP problems are typically solved using the branch-and-bound technique [50], a non-iterative approach that has been implemented in several libraries. The idea behind MILP has been applied to solve a variety of optimization problems, such as traveling salesman, scheduling, and generalized assignment [51,52,53]. Previously used for DA, MILP is implemented in this study using the lp_solve library [54]. The nature of MILP means that we have to combine our objective functions into one function and add more constraints for MILP to work:

(4a)maximize   ∑i=1n∑j=1mlog(1+rji∗cji)−∑j=1maj(4b)subject to:∑i=1ncji≥aj  ∀j∈AP(4c)    ∑i=1ncji≤n∗aj ∀j∈AP(4d)     aj∈{0,1}   ∀j∈AP

The objective function 4a is actually the combined form of two objective functions, (1a) and (1b), previously defined in Section 4. The purpose of both constraints, (4b) and (4c), is to ensure that $a_{j}=min\left(\sum_{i=1}^{n}c_{ji},1\right)$. This means that if $ap_{j}$ has no stations associated with it, $a_{j}=0$, otherwise $a_{j}=1$. This transformation allows the DA problem to be solved by lp_solve.

### 5.6. Maximum Network Throughput

In order to understand the impact of the association order, we run hundreds of repetitions with different orders using the same stations based on location and demand (using the same random seed). For the proposed DA algorithm and MILP, no repetition is performed because these approaches do not depend on the association order.

**Figure 10:**
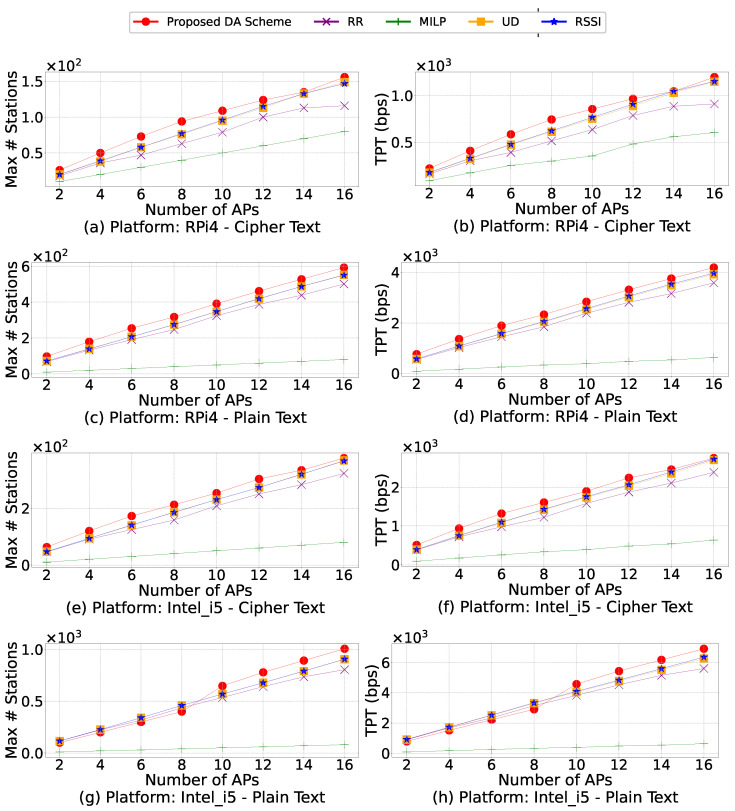
Simulation results for all five algorithms on RPi4 and Intel_i5 platforms for cipher text and plain text. These plots show how each algorithm performs in terms of the network throughput (TPT), which is measured in bits per second (bps) and the maximum number of devices supported.

In order to identify the maximum throughput supported in this network, we deploy different numbers of APs and run all five algorithms while gradually increasing the number of stations until no solution is found (the APs’ maximum capacity is reached). The higher this upper bound, the better the algorithm. In some cases, a particular order cannot satisfy the AP’s constraints (security or capacity). When this occurs in repetition, the number of stations is reduced, and the association is attempted again until a solution is found. For this experiment, which is demonstrated by Figure 10, we deploy a range of APs from two to 16 in increments of two APs in the same area in order to evaluate the throughput and the maximum number of stations supported.

In Figure 10, simulation results for all five algorithms on different platforms with plain and cipher messages are shown. Since the evolutionary mechanisms of the proposed DA scheme allow it to converge to optimal solutions, it surpasses other algorithms in our experiments. Unlike the proposed DA scheme, the RSSI, RR, and UD algorithms highly depend on the association order. As previously mentioned, since the data for these approaches consists of hundreds of repetitions, changing the association order can affect the final solutions of RSSI, RR, and UD. It is known that in some repetitions, a particular association order may lead to no valid solutions, which requires reducing the number of stations until an association is possible. Therefore, these approaches are not as robust as the proposed DA scheme. Out of all these algorithms, MILP performs the worst. This is because MILP requires heavy computation to identify the optimal solution, and the computing power of our testbed limits its performance. Furthermore, there is a strong correlation between network throughput and the maximum number of stations supported, which can be explained by Equation (1a). Nevertheless, the proposed DA scheme can always provide better throughput than other comparison algorithms for any given number of stations.

### 5.7. Minimum Number of APs

**Figure 11:**
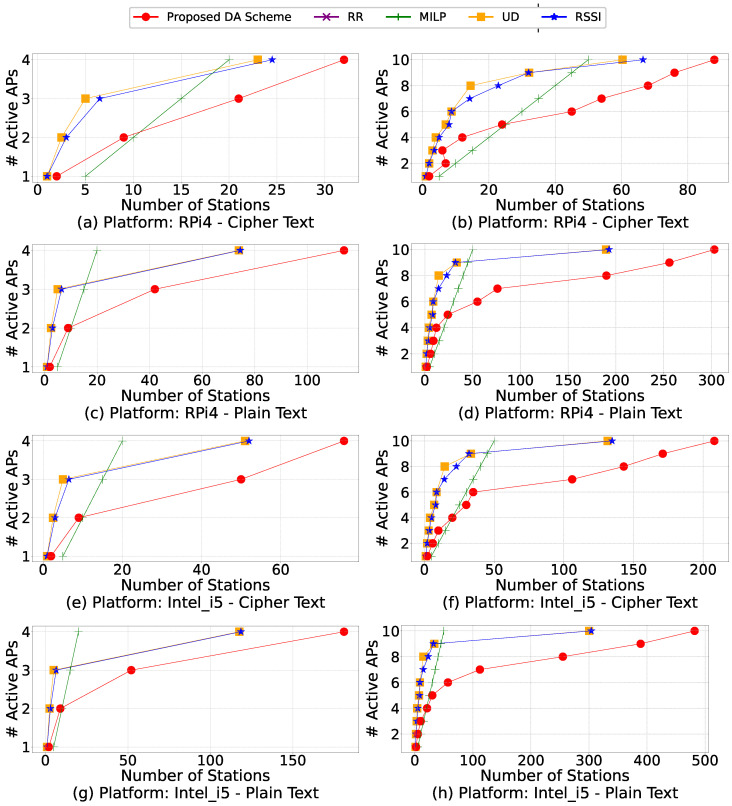
Number of active APs required by a fixed amount of network throughput. Four algorithms are implemented on RPi4 and Intel_i5 platforms across two scenarios: cipher text and plain text. The number of active APs could be lower than the total number of APs available, as some algorithms do not need all APs to be able to support a particular number of stations.

Another experiment is performed to find the minimum number of active APs, which is the lower bound needed to support a fixed number of stations. Without losing generality, we assume all stations generate an identical amount of traffic. Thus, we can represent a fixed amount of overall network throughput by fixing the number of stations. In this experiment, as demonstrated by Figure 11, we simulate a small and a large network by deploying four and 10 APs in a given area, respectively. Please note that these are the total number of available APs. Different algorithms will end up employing a different number of APs to satisfy the overall throughput requirements. We consider the algorithm that yields the smallest number of active APs as the best one.

Figure 11 shows the performance of different algorithms in terms of the number of active APs required to support a fixed number of stations. RR is not shown here as it always uses all the APs in the network. The left column of Figure 11 involves only four total available APs, representing smaller networks, and the right column of Figure 11 involves ten total available APs, representing larger networks.

When the number of stations increases, the number of required APs increases in a discrete way. For example, adding five more stations may not require any extra AP, while adding six more stations may suddenly require an extra AP. Therefore, although we run each specific number of stations in our experiments, in Figure 11, we only mark the number of stations that leads to an increase in the number of active APs.

Based on Figure 11, we observe that to support a fixed number of stations, the proposed DA scheme requires fewer APs, especially for large networks with more stations. For example, in Figure 11a, when there are 20 stations, the proposed DA scheme can associate them with two APs. In contrast, the RSSI and UD algorithms need three APs, while the MILP algorithm requires four APs. The proposed DA scheme also shows superiority when there are ten APs. Taking Figure 11b as an example, when there are 40 stations, the proposed DA scheme can associate all of them with five APs, while the MILP requires eight APs, and the RSSI and UD require nine APs. In the case of MILP, when more stations are added, the required number of active APs increases linearly, which is undesired. As discussed before, this is because MILP requires heavy computation to identify the optimal solution, and its performance is limited by the computing power of our testbed.

As shown in the results, the proposed DA scheme outperforms all the other schemes; unlike the remaining schemes, it provides flexibility in evaluating the feasibility of each potential solution during every iteration, leading to a better DA solution and less resource consumption for the network. The GA can be further fine-tuned through various parameters as shown in Table 6. It is relatively easy to modify the parameters to ensure quick convergence to a solution depending on the size of the network, which helps to reasonably limit computational complexity in a real-world deployment. The real benefit of the proposed DA scheme is its ability to deliver a macro-scale reduction of resource consumption for the entire network. The proposed DA scheme could be executed on either a regular machine (Intel_i5) or a resource-constrained device (RPi4).

The offloading framework and the DA scheme complement each other. In a real-world deployment, the offloading framework’s findBestAP functionality calls the proposed DA scheme to identify the best AP for the current station. By periodically monitoring the network and reassigning the stations by the proposed DA scheme, as mentioned in Figure 4, the proposed framework is able to ensure that the network achieves high throughput and the number of active APs is reduced. Combining the offloading framework and the proposed DA scheme should enable a network to handle a large number of stations and APs smoothly and securely.

## 6. Conclusions

This work proposed a comprehensive framework for resource-constrained IoT stations to offload the heavy burden of TLS connections to WiFi APs. We focused on large-scale scenarios where multiple APs are available, and each IoT station must be associated with the most appropriate AP. We model the device association problem as a multi-objective optimization issue that maximizes network throughput while minimizing the number of APs. Experimental results validate the offloading framework’s significant overhead savings compared to the conventional approach of using TLS and the proposed DA scheme’s superiority over other comparison schemes. The proposed framework can be adopted in settings with a large number of resource-constrained IoT stations that transfer data through a secured channel, such as industrial IoT or smart cities. The proposed framework can ensure secure communication while enhancing the lifespan of stations.

## Figures and Tables

**Table 1:** An overview of the related works on offloading schemes, cryptographic optimizations, and device association (DA). ‘DA factors’ refer to the input an implementation needs to perform DA (only applicable for works covering DA). E: Experimental. S: Simulated. PO: Partial offloading. FO: Full offloading. CO: Cryptographic optimization. CEN: Centralized. DIST: Distributed.

Reference	E/S	Category	DA Factors	Architecture
[8]	E	PO, CO	N/A	N/A
[9]	E	FO	N/A	N/A
[10]	S	FO	N/A	N/A
[11]	E	CO	N/A	N/A
[12]	E	CO	N/A	N/A
[13]	E	CO	N/A	N/A
[14]	E	CO	N/A	N/A
[15]	S	DA	Throughput, load balancing	CEN
[16]	S	DA	Network utility, user-AP airtime	CEN
[17]	S	DA	Channel assignment, multicast.	CEN
[18]	S	DA	Load balancing, throughput	CEN
[19]	S	DA	Load balancing, energy savings, throughput	CEN
[20]	S	DA	Load balancing, energy savings	CEN
[21]	S	DA	Throughput, load balancing, free air time	CEN
[22]	S	DA	Throughput	CEN
[23]	S	DA	Throughput	DIST
[24]	S	DA	Fairness, throughput	DIST
[25]	S	DA	Throughput	DIST
[26]	S	DA	Channel utilization	DIST

**Table 2:** Physical meanings of key symbols.

Symbol	Definition
AP	Set of *m* access points $\left\{ap_{1},ap_{2},\cdots ,ap_{m}\right\}$
${\mathrm{AP}}_{j}$	Set of stations associated with access point $ap_{j}$
STA	Set of *n* stations $\left\{sta_{1},sta_{2},\cdots ,sta_{n}\right\}$
${\mathrm{SNR}}_{ji}$	Signal to noise ratio between station $sta_{i}$ and access point $ap_{j}$
$c_{ji}$	Binary variable indicating 1 if station $sta_{i}$ is associated with $ap_{j}$, 0 otherwise
$g_{iu}$	Binary variable. If station $sta_{i}\in {\mathrm{AP}}_{j}$ interferes with station $sta_{u}\in {\mathrm{AP}}_{z}$, j≠z, and both stations are transmitting concurrently, then $g_{iu}$ equals 1, otherwise, $g_{iu}$ equals 0
$a_{j}$	Binary variable indicating 1 if there is one or more users associated with $ap_{j}$, 0 otherwise
$r_{ji}$	Rate from station $sta_{i}$ to access point $ap_{j}$
$m_{i}^{k}$	Number of messages of type k∈K ( size $m^{k}$ ) that station $sta_{i}$ sends every second
*f*	A placeholder function name which could be substituted for either $f_{c}$ or $f_{sc}$
$f_{c}$	A function returning the number of messages processed with non-secure communication every second per core for a given size of message
$f_{sc}$	A function returning the number of messages processed with secured communication (featuring encryption and decryption) every second per core for a given size message
$R_{0}$	Minimum AP signal strength needed so that a station can connect with it
$sl_{i}$	Security level assumed by device $sta_{i}$
$\psi_{ji}$	Received signal strength at access point $ap_{j}$ transmitted by the antenna of station $sta_{i}$ from a distance *d*
B	Channel bandwidth
$R_{ji}$	Received signal strength at station $sta_{i}$ that is transmitted from access point $ap_{j}$
$P_{0}$	Carrier sensing threshold
$\sigma^{2}$	Additive Gaussian white noise
$S_{0}$	Security threshold

**Table 3:** Summary of the specifications of hardware platforms used in this work.

Platform	CYW43907 (CYW)	Raspberry Pi 4 B (RPi4)	Intel Core i5 (Intel_i5)
MCU	ARM Cortex R4	ARM Cortex A72	Intel Core i5
Word Size	32-bit	64-bit	64-bit
RAM	2 MB	2 GB	4 GB
Clock Frequency	320 MHz	1.5 GHz	2.4 GHz
WiFi Standards	802.11b/g/n	802.11b/g/n/ac	802.11b/g/n/ac
On-chip Crypto Core	Available	Not Available	Available

**Table 4:** Summary of resource consumption values. This table presents the effect of offloading on resource saving. Values shown in this table are the average of 10 iterations for each task. Confidence intervals are presented.

Task	HS	HS and Records	Encrypted Messages
Energy (J)	2.538 ± 0.195	2.729 ± 0.365	0.173 ± 0.060
Duration (s)	20.408 ± 1.554	21.005 ± 2.868	1.326 ± 0.435

**Table 5:** Summary of coefficients for $f_{sc}$ (cipher text) and $f_{c}$ (plain text) functions. Both regression functions are under the form $F=\beta_{1}+m\beta_{2}+m^{2}\beta_{3}$.

Platform	Function	$\beta_{1}$	$\beta_{2}$	$\beta_{3}$
Intel_i5	$f_{sc}$	2342.165	0.090	$-9.751\times 10^{-5}$
Intel_i5	$f_{c}$	5701.493	−0.230	$-2.117\times 10^{-4}$
RPi4	$f_{sc}$	997.379	−0.050	$6.856\times 10^{-6}$
RPi4	$f_{c}$	3945.306	−0.467	$-1.409\times 10^{-4}$

**Table 6:** Summary of GA parameters and the values used in our implementation.

Symbol	Definition	Actual Value
μ	Number of individuals to select for the next generation	25
λ	Number of children to produce at each generation	50
*cxpb*	The probability that an offspring is produced by crossover	0.75
*mutpb*	The probability that an offspring is produced by mutation	0.2
*ngen*	Number of generations	200

## Data Availability

This project’s repository can be accessed at https://github.com/ranofal/offloading_framework_DA_simulation_AI on (24 September 2021).
